# Ibuprofen mediates histone modification to diminish cancer cell stemness properties via a COX2-dependent manner

**DOI:** 10.1038/s41416-020-0906-7

**Published:** 2020-06-12

**Authors:** Wenzhi Shen, Xiaoyuan Zhang, Renle Du, Wenjuan Gao, Juan Wang, Yonghua Bao, Wancai Yang, Na Luo, Jianjun Li

**Affiliations:** 1 Department of Pathology and Institute of Precision Medicine, Jining Medical University, Jining, 272067 China; 2 Institute of Breast Research, Jining Medical University, Jining, 272067 China; 3 Department of Immunology, School of Medicine, Nankai University, Tianjin, 300071 China

**Keywords:** Cancer therapy, Cancer stem cells

## Abstract

**Background:**

The anticancer potential of ibuprofen has created a broad interest to explore the clinical benefits of ibuprofen in cancer therapy. However, the current understanding of the molecular mechanisms involved in the anticancer potential of ibuprofen remains limited.

**Methods:**

Cancer stemness assays to validate ibuprofen function in vitro and in vivo. Histone modification assays to check the effect of ibuprofen on histone acetylation/methylation, as well as the activity of HDAC and KDM6A/B. Inhibitors’ in vivo assays to evaluate therapeutic effects of various inhibitors’ combination manners.

**Results:**

In our in vitro studies, we report that ibuprofen diminishes cancer cell stemness properties that include reducing the ALDH + subpopulation, side population and sphere formation in three cancer types. In our in vivo studies, we report that ibuprofen decreases tumour growth, metastasis and prolongs survival. In addition, our results showed that ibuprofen inhibits inflammation-related stemness gene expression (especially ICAM3) identified by a high-throughput siRNA platform. In regard to the underlying molecular mechanism of action, we report that ibuprofen reduces HDACs and histone demethylase (KDM6A/B) expression that mediates histone acetylation and methylation, and suppresses gene expression via a COX2-dependent way. In regard to therapeutic strategies, we report that ibuprofen combined HDAC/HDM inhibitors prevents cancer progression in vivo.

**Conclusions:**

The aforementioned findings suggest a molecular model that explains how ibuprofen diminishes cancer cell stemness properties. These may provide novel targets for therapeutic strategies involving ibuprofen in the prevention of cancer progression.

## Background

The cancer stem cell (CSC) is the chief culprit in tumour initiation and malignancy whereby the maintenance of cancer cell stemness largely depends on the surrounding environment or also called the ‘niche’.^1–3^ Although the highly tumorigenic CSCs play a role in tumour initiation/metastasis, and might therefore be a good clinical therapy target, CSCs unfortunately demonstrate a relative resistance to conventional chemotherapy and radiotherapy. In addition, tumour-associated inflammation factors within the tumour niche play a pivotal role in the maintenance of cancer cell stemness and the resultant tumour initiation/malignancy.^4,5^ Our previous work presented a medium-throughput siRNA screen platform to identify inflammation genes that regulate cancer cell stemness, and obtained several novel candidates.^6^ Agents that target these genes may inhibit both inflammation and cancer cell stemness at the same time.

Ibuprofen (a non-steroidal anti-inflammatory drug) is commonly used for treating pain, fever and inflammation. Recent observational and epidemiological studies have shown that regular, prolonged use of ibuprofen reduces the risk for several cancers (e.g., colorectal, breast, cervical, gastric, lung cancers and head and neck cancer).^7–14^ Although the benefits of ibuprofen for cancer patients have been appreciated, the mechanism remains unclear. Previous studies attribute the anticancer potential of NSAID like aspirin to the inhibition of cyclooxygenase-2 (COX2), which is upregulated in various cancer cells.^15,16^ Of note, an increasing body of evidence suggests that aspirin may exhibit anticancer effects in a COX-independent manner. However, the role of COX expression in ibuprofen-mediated cancer inhibition remains unclear.

Histone modification is a reversible process mediated by the epigenetic enzymes.^17,18^ Histone methylation and acetylation are two important chemical modifications that act in transcriptional activation/inactivation, chromosome packaging and DNA damage/repair.^19,20^ Histone demethylases (HDMs) and histone deacetylases (HDACs) are the key enzymes that remove methyl and acetyl groups, respectively, to regulate gene transcription. In this regard, NSAID like aspirin affects HDAC expression and suppresses progression of some cancers.^21–23^ However, the role of ibuprofen on histone modification and the specific mechanisms involved remain unclear. Thus, we studied the role of ibuprofen on histone methylation and acetylation, and the attendant effects on cancer cell stemness and progression.

Here, by investigating the role of ibuprofen on cancer cell stemness and progression, we found that ibuprofen restrains cancer cell stemness properties that include reducing ALDH + subpopulation, side population and sphere formation in three cancer types in vitro. Furthermore, ibuprofen inhibits tumour growth, metastasis and prolongs survival in vivo. In addition, ibuprofen was proved to inhibit inflammation-related stemness genes, especially ICAM3 that we screened by high-throughput siRNA platform. Exploration of the underlying mechanism demonstrated that ibuprofen reduced HDACs and histone demethylase (KDM6A/B) expression to mediate histone 3 methylation and acetylation to suppress gene expression via a COX2-dependent manner. As the therapy strategies, ibuprofen combined HDAC/HDM inhibitors, and restrained cancer progression in vivo. Our research revealed the promising mechanism or strategies of ibuprofen in the prevention of cancer progression.

## Methods

### Cytotoxicity assay

Ibuprofen was purchased in Sigma (cat. I4883) and dissolved in DMSO. MDA-MB-231, A549 and HepG2 cells were cultured in a 96-well plate and treated with various concentrations of ibuprofen for 24 h. Cell activity was tested by applying CCK8 kit (Dojindo, China) following the manufacturer’s instructions.

### Aldefluor assay

The Aldefluor assay kit (STEMCELL Technologies, Vancouver, Canada) was used to measure ALDH enzymatic activity in three cancer cell lines (MDA-MB-231, A549 and HepG2). In brief, 2.5 × 10^5^ cells were suspended in Aldefluor assay buffer containing ALDH1 substrate and incubated for 60 min at 37 °C. Cells treated with the specific ALDH inhibitor DEAB, served as the negative control. Stained cells were analysed on BD FACS Calibur flow cytometer (BD Biosciences, San Jose, CA). Data analysis was performed using Flowjo software (Tree Star, Inc., Ashland, OR).

### Side-population assay

In total, 231, A549 and HepG2 cells treated with ibuprofen were harvested and resuspended in pre-warmed staining buffer (PBS buffer added 2% FBS) at a density of 1.0 × 10^6^ cells/ml. Hoechst 33342 dye was added at a final concentration of 7 µg/ml (231), 8 µg/ml (A549) and 10 µg/ml (HepG2) in the presence or absence of 10 µM fumitremorgin C (FTC). The following steps were described previously.^6,24^

### QPCR assay

Total RNAs of cells were extracted by TRIzol reagent (Cat. #15596-018, Invitrogen Inc., Carlsbad, CA) and then reverse-transcribed into cDNAs. Real-time PCR was performed in 20-μl reaction volumes by using TransStart Green qPCR SuperMix Kit (TransGen Biotech, Beijing, China, PR). The 2^–ΔΔCt^ method was used to determine the relative mRNA folding changes. Statistical results were averaged from three independent experiments performed in triplicate. The specific primer sequences are summarised in Supplementary Table 1.

### Sphere-formation assay

The sphere-formation assay steps were described previously.^6^

### Animal study

Female Balb/c mice at 6–8 weeks were separated randomly into several groups (*n* ≥ 5). In total, 5 × 10^4^ 4T1-luci cells were inoculated s.c. into each mouse at the right axilla. For lung metastasis assay, at 7 days after injection, mice were treated with ibuprofen 20 mg/kg, ibuprofen 40 mg/kg every 3 days and DMSO used as the control. For chemoresistance assay, at 7 days after injection, mice were first treated with cisplatin (2.5 mg/kg, 0.9% NaCl dissolved), then treated with ibuprofen 10 mg/kg, ibuprofen 20 mg/kg every 3 days until the mice were dead finally. DMSO was used as the control.

Female nude mice at 6–8 weeks were separated randomly into several groups (*n* ≥ 5). In total, 2 × 10^6^ ALDH + or ALDH− cells were inoculated s.c. into each mouse at the right axilla. For lung metastasis assay, at 12 days after injection, mice were treated with ibuprofen 20 mg/kg, ibuprofen 40 mg/kg every 3 days and DMSO used as the control. For chemoresistance assay, at 12 days after injection, mice were first treated with cisplatin (2.5 mg/kg), then treated with ibuprofen 10 mg/kg, ibuprofen 20 mg/kg every 3 days until 24 days. The mice were sacrificed by isoflurane anaesthesia treatment. DMSO was used as the control.

NOD/SCID mice at 6–8 weeks were separated randomly into several groups (*n* ≥ 5). In total, 3 × 10^6^ A549-luci cells were inoculated s.c. into each mouse. For inhibitor treatment assay, 21 or 23 days after tumour cells injection, mice were first treated with ibuprofen 10 mg/kg or HDAC inhibitor (Trichostatin A (TSA), 0.5 mg/kg), KDM6A/B inhibitor (GSK J1, 100 mg/kg), then every 3 days treated until the mice were dead finally. DMSO was used as the control.

Tumour volume (mm^3^) was measured with callipers and calculated by using the standard formula: length × width^2^/2. The individual measuring the mice was unaware of the identity of the group measured. Animal use complied with Nankai University and Jining Medical University Animal Welfare Guidelines.^25^

### Western blotting

The western blot steps were described previously.^6,26^ The special primary antibodies used in this assay are listed in Supplementary Table 2.

### Immunofluorescence

The immunofluorescence assay was described previously.^6,25^

### TUNEL staining

Paraffin-embedded tissue slides were prepared from the tumour xenografts’ DeadEnd^TM^ Fluorometric TUNEL System kit (Promega) that was applied for TUNEL staining. The experiment procedure was performed according to the paper instruction. 4,6-Diamidino-2-phenylindole was used to stain the nuclei, and the tissue slides were subjected to Olympus BX51 Epi-fluorescent microscopy under a 40× objective (FV1000-IX81, Olympus Microsystems, Shanghai, China).

### Chromatin immunoprecipitation assay

The assay was performed with an EZ-Zyme Chromatin Prep Kit (Millipore), according to the manufacturer’s protocol. Anti-histone 3 modification markers’ antibodies were used to precipitate DNA cross-linked with histone 3 modification markers, respectively, and normal rabbit IgG was used in parallel as a control. Enriched DNA was then used as a template to assess the binding intensity of histone 3 modification markers to putative binding sites in the ICAM3 promoter. Primers used in this assay are listed in Supplementary Table S2.

### Immunohistochemistry

Immunohistochemistry was performed on tumour tissue sections from the mice. Primary antibodies were raised against the target proteins at a 1:100 dilution overnight. The expression levels of the proteins were evaluated according to the percentage of positive cells in each tumour tissue section. The images were recorded by Olympus BX51 Epi-fluorescent microscopy under a 20× or 40× objective (Olympus Co, Tokyo, Japan).^27^

### HDAC activity assay

The HDAC activity in cancer cells was evaluated using an Epigenase HDAC Activity/Inhibition Direct Assay Kit (Epigentek, Farmingdale, NY) following the manufacturer’s instruction. The nuclear extract was prepared by using NE-PER™ Nuclear and Cytoplasmic Protein Extraction Reagents (ThermoFisher Scientific) and quantitated. The relative HDAC activity was calculated as the ratio of the HDAC activity of the ibuprofen group compared with that of the control (DMSO) group.

### Statistical analysis

All data were analysed using GraphPad Prism5 software (GraphPad Software, San Diego, CA, USA). Values were expressed as means ± SEM. *P* values were calculated using a two-tailed Student’s *t* test (two groups) or one-way ANOVA (more than two groups), unless otherwise noted. A value of *P* < 0.05 was used as the criterion for statistical significance. *Indicates significant difference with *P* < 0.05, **indicates significant difference with *P* < 0.01, ***indicates significant difference with *P* < 0.001.^6,28^

## Results

### Ibuprofen diminishes cancer cell stemness properties in vitro

In order to establish the proper working concentrations of ibuprofen in various cancer cells, we determined the IC_50_ of ibuprofen in A549 lung cancer cells, MDA-MB-231 breast cancer cells and HepG2 liver cancer cells using a cytotoxicity assay. Our results showed a 3.0 mM IC_50_ in A549 lung cancer cells, a 1.8 mM IC_50_ in MDA-MB-231 breast cancer cells and a 1.2 mM IC_50_ in HepG2 liver cancer cells (Supplementary Fig. S1). Based on the IC_50_, we chose working concentrations of 0, 0.5 and 1 mM ibuprofen for three types of cancer cells in our studies.

**Fig. 1:**
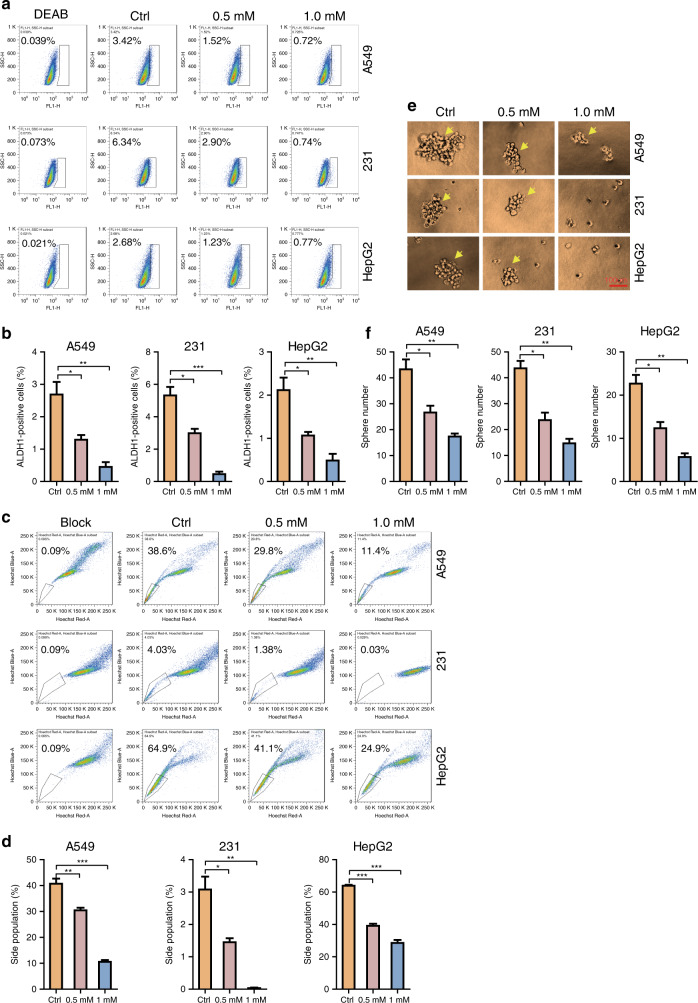
Ibuprofen restrains cancer cell stemness properties in vitro. **a** ALDH staining assay was performed to check ALDH + sub-population percentage in the three cancer cell lines with or without ibuprofen treatment. **b** Statistic results of ALDH sub-population percentage were shown. **c** Side-population assay was performed to detect the SP percentage in three cancer cell lines with or without ibuprofen treatment. **d** Statistic results of SP percentage were shown. **e** Sphere-formation assay was performed to check the cell-sphere-formation ability in the three cancer cell lines with or without ibuprofen treatment, scale bar: 100 µm. **f** Statistic results of sphere amounts were shown. All data shown are representative of at least three experiments.

In order to determine the in vitro effects of ibuprofen on cancer cell stemness, we investigated ALDH + sub-population changes in A549 lung cancer cells, MDA-MB-231 breast cancer cells and HepG2 liver cancer cells using the ALDH staining assay. Our results indicated that the ALDH + subpopulation decreases in the ibuprofen-treated groups versus controls (Fig. 1a, b). In order to determine the effects of ibuprofen on cancer cell stemness, we next investigated the changes in the side population in the three cancer cell lines using the side-population assay. Our results indicated that the side population decreases in the ibuprofen-treated groups versus controls (Fig. 1c, d). In order to determine the effects of ibuprofen on cancer cell stemness, we next investigated the changes in cell-sphere formation in the three cancer cell lines using the sphere- formation assay. Our results showed that sphere formation decreases in the ibuprofen-treated groups versus controls (Fig. 1e, f).

To further determine the effects of ibuprofen on cancer stem cells and normal cancer cells, we sorted the ALDH + cells (CSC) and ALDH− cells (normal cancer cell) from 231 and A549 cells and treated ibuprofen, respectively (Supplementary Fig. S2A), and checked the stemness markers’ expression by western blot. The results showed that ibuprofen reduced the stemness marker (ALDH1A1, SOX2, OCT4 and NANOG) expression in both ALDH + and ALDH− cells (Supplementary Fig. S2B).

Collectively, the above-mentioned findings suggest that ibuprofen diminishes cancer cell stemness properties with the CSC non-specific target way in vitro.

### Ibuprofen diminishes cancer cell metastasis and stemness properties in vivo

**Fig. 2:**
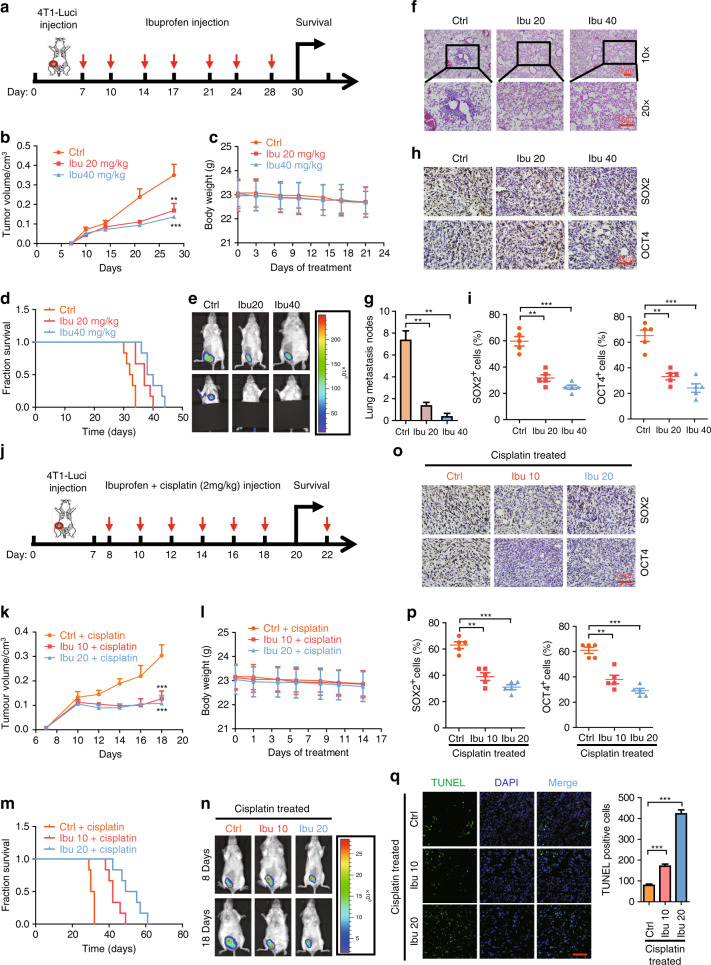
Ibuprofen suppresses cancer cell metastasis and stemness in vivo. **a** Schema of the metastasis model established by subcutaneous implantation of 4T1-luci cells into the fourth pair of mammary fat pad of BALB/c mice. **b** Tumour growth curve of 4T1-luci with or without ibuprofen treatment. **c** The body weight of BALB/c mice in the course of ibuprofen treatment. **d** The survival curve of BALB/c mice inoculated with 4T1-luci with or without ibuprofen treatment. **e** The representative luciferase images showing the 4T1-luci tumours at the primary site and lung metastasis sites with or without ibuprofen treatment. **f** Representative H&E staining images of 4T1-luci tumour metastasis to the lung with or without ibuprofen treatment, scale bar: 50 µm. **g** Statistic results of metastasis loci of 4T1-luci tumour metastasis to the lung with or without ibuprofen treatment. **h** Immunohistochemistry staining of SOX2 and OCT4 in 4T1-luci primary tumours with or without ibuprofen treatment. Representative images with 20× magnification were shown, scale bar: 50 µm. **i** Statistic results of SOX2- or OCT4-positive cells in 4T1-luci primary tumours with or without ibuprofen treatment. **j** Schema of the chemoresistance model established by subcutaneous implantation of 4T1-luci cells into the fourth pair of mammary fat pad of BALB/c mice. **k** Tumour growth curve of 4T1-luci with or without ibuprofen treatment in the presence of cisplatin. **l** The body weight of BALB/c mice in the course of ibuprofen treatment in the presence of cisplatin. **m** The survival curve of BALB/c mice inoculated with 4T1-luci with or without ibuprofen treatment in the presence of cisplatin. **n** The representative luciferase images showing 4T1-luci tumours at the primary sites with or without ibuprofen treatment on day 7 (before cisplatin administration) and day 18 (after cisplatin administration). **o** Immunohistochemistry staining of SOX2 and OCT4 in 4T1-luci primary tumours with or without ibuprofen treatment in the presence of cisplatin, scale bar: 50 µm. Representative images with 20× magnification were shown. **p** Statistic results of SOX2- or OCT4-positive cells in 4T1-luci primary tumours with or without ibuprofen treatment in the presence of cisplatin. All data shown are representative of three experiment repeats. **q** Representative images showing TUNEL staining of tumour tissue from each group. Scar bar, 100 μm.

In order to determine the effects of ibuprofen on cancer cell metastasis and stemness in vivo, we implanted 4T1-luciferase cells into the fourth fat pad of female Balb/c mice. Seven days after implantation, we IP-injected the mice with 20 mg/kg ibuprofen, 40 mg/kg ibuprofen or DMSO (control group) two times per week (Fig. 2a). Our results showed that tumour volume decreases in the ibuprofen-treated groups versus the control (Fig. 2b). However, we found that the body weight did not change in the ibuprofen-treated groups versus the control (Fig. 2c). In addition, we found that the survival time increases in the ibuprofen-treated groups versus control (Fig. 2d). With respect to the effect of ibuprofen on cancer cell metastasis, we found that lung metastasis decreases in ibuprofen-treated groups versus the control (Fig. 2e–g). With respect to the effect of ibuprofen on cancer cell stemness properties, we found that the immunocytochemical staining of SOX2 and OCT4 stemness markers decreases in the ibuprofen-treated groups versus DMSO controls (Fig. 2h, i).

To further verify the effects of ibuprofen on cancer stem cells and normal cancer cells in vivo, we sorted the ALDH + cells (CSC) and ALDH− cells (normal cancer cell) from 231 cells and injected the nude mice subcutaneously (Supplementary Fig. S2C). About 12 days after implantation, we IP-injected the mice with 20 mg/kg ibuprofen, 40 mg/kg ibuprofen or DMSO (control group) every 3 days (Supplementary Fig. S2D). The results showed that tumour growth and volume decreased in the ibuprofen-treated groups versus the control in both ALDH + and ALDH− cell groups (Supplementary Fig. S2E, S2H).

Together, the above-mentioned findings suggest that ibuprofen diminishes cancer cell metastasis and stemness properties with the CSC non-specific target way in vivo.

### Ibuprofen reduces cancer cell chemoresistance in vivo

As chemoresistance was a very important feature of cancer cell stemness in the clinic, cancer cells with stemness property were resistant to a chemoreagent like cisplatin therapy. In order to determine the effects of ibuprofen on cancer cell chemoresistance in vivo, we implanted 4T1-luciferase cells into the fourth fat pad of female Balb/c mice. About 8 days after implantation, we IP-injected the mice with 2 mg/kg cisplatin + 10 mg/kg ibuprofen, 2 mg/kg cisplatin + 20 mg/kg ibuprofen or 2 mg/kg cisplatin + DMSO (control group) every 3 days (Fig. 2j). Our results showed that tumour volume and tumour growth speed decrease in the cisplatin/ibuprofen-treated versus the cisplatin/DMSO control (Fig. 2k & n). However, we found that the body weight did not change in the cisplatin/ibuprofen-treated versus the cisplatin/DMSO control (Fig. 2l). In addition, we found that the survival time increases in the cisplatin/ibuprofen-treated groups versus the cisplatin/DMSO control (Fig. 2m). Consistent with the in vitro cell apoptosis assay, we also found that the cell apoptosis (TUNEL + cells) was increased in the cisplatin/ibuprofen-treated versus the cisplatin/DMSO control (Fig. 2q). With respect to the effect of ibuprofen on cancer cell stemness properties, we found that the immunocytochemical staining of SOX2 and OCT4 stemness markers decreases in the cisplatin/ibuprofen-treated groups versus cisplatin/DMSO control (Fig. 2o, p).

To further identify the effects of ibuprofen on cancer stem cells and normal cancer cells’ chemoresistance in vivo, we sorted the ALDH + cells (CSC) and ALDH− cells (normal cancer cell) from 231 cells, and injected the nude mice subcutaneously (Supplementary Fig. S2C). About 12 days after implantation, we IP-injected the mice with 2 mg/kg cisplatin + 10 mg/kg ibuprofen, 2 mg/kg cisplatin + 20 mg/kg ibuprofen or 2 mg/kg cisplatin + DMSO (control group) every 3 days (Supplementary Fig. S2F). The results showed that tumour volume and tumour growth speed decrease in the cisplatin/ibuprofen-treated versus the cisplatin/DMSO control in both ALDH + and ALDH− cell groups (Supplementary Fig. S2G, S2H).

Thus, the above-mentioned findings suggest that ibuprofen reduces cancer cell chemoresistance with the CSC non-specific target way in vivo.

### Ibuprofen inhibits the expression of inflammation-related stemness genes in vitro and in vivo

**Fig. 3:**
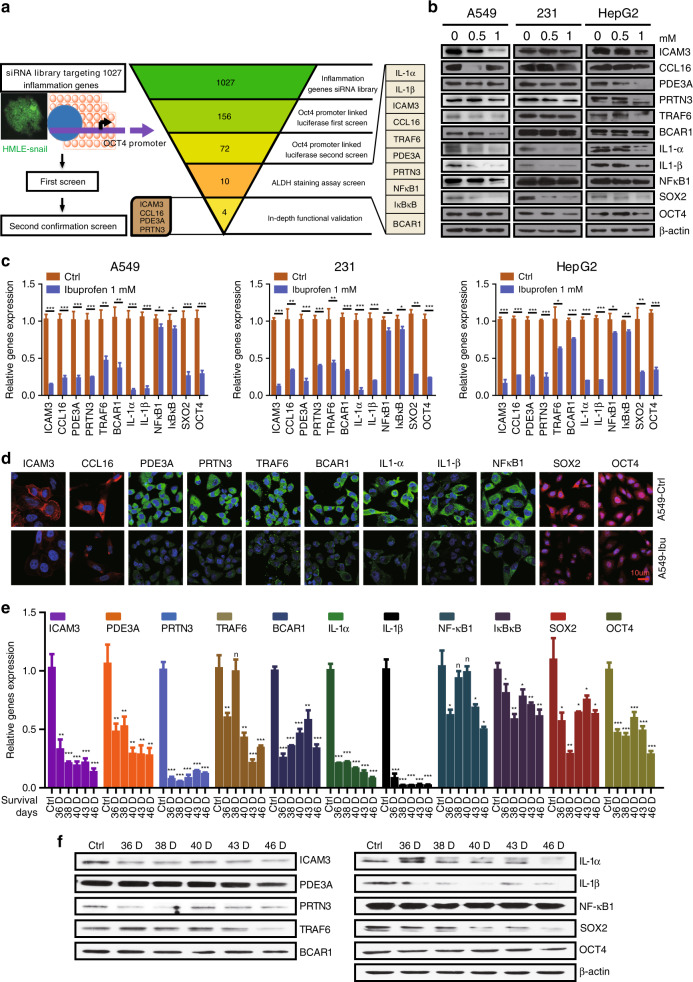
Ibuprofen inhibits the expression of inflammation-related stemness genes in vitro and in vivo. **a** Schematic representation of the siRNA screen (left). Summary of the results from the RNAi screen (right). **b** Western blot examining the expression of inflammatory candidates and stemness proteins (SOX2 and OCT4) in A549, MDA-MB-231 and HepG2 cells with or without ibuprofen treatment. **c** Quantitative PCR examining the mRNA expression of inflammatory candidates and stemness genes (SOX2 and OCT4) in A549, MDA-MB-231 and HepG2 cells with or without ibuprofen treatment. **d** Immunofluorescence staining of inflammatory candidates and stemness genes (SOX2 and OCT4) in A549, MDA-MB-231 and HepG2 cells with or without ibuprofen treatment, scale bar: 10 µm. **e** Quantitative PCR examining the mRNA expression of inflammatory candidates and stemness genes (SOX2 and OCT4) in 4T1-luci tumours separated from BALB/c mice treated with ibuprofen for different survival days. **f** Western blot examining the expression of inflammatory candidates and stemness genes (SOX2 and OCT4) in 4T1-luci tumours separated from BALB/c mice treated with ibuprofen for different survival days. All data shown are representative of three experiment repeats.

Our previously published report established a medium-throughput siRNA screening platform that identifies inflammation genes that regulate cancer cell stemness. Specifically, we identified several novel candidate genes that decrease OCT4 expression and the ALDH + subpopulation, both of which characterise stemness (Fig. 3a).

In order to determine whether ibuprofen decreases the expression of these novel candidate genes to further diminish cancer cell stemness, we investigated the expression of novel candidate genes and stemness markers (SOX2 and OCT4) in A549 lung cancer cells, MDA-MB-231 breast cancer cells and HepG2 liver cancer cells using western blot. Our results showed that ICAM3, CCL16, PDE3A, PRTN3, SOX2 and OCT4 protein expression decreases in the ibuprofen-treated groups versus controls (Fig. 3b). We also found that ICAM3, CCL16, PDE3A, PRTN3, TRAF6, BCAR1, IL-1α, IL-1β, NFκB1, IκBκB, SOX2 and OCT4 mRNA expression decreases in the ibuprofen-treated group versus control (Fig. 3c). Moreover, ICAM3, CCL16, PDE3A, PRTN3, TRAF6, BCAR1, IL-1α, IL-1β, NFκB1, SOX2 and OCT4 decreases the protein expression as indicated by immunofluorescence staining in the ibuprofen-treated MDA-MB-231 breast cancer cells versus the control (Fig. 3d).

To further investigate the role of ibuprofen on novel candidate genes in cancer stem cells and normal cancer cells, we checked these genes’ expression in ALDH + and ALDH− cells treated with ibuprofen by western blot. The results showed that these genes’ expression decreased in both ALDH + and ALDH− cells (Supplementary Fig. S3A).

In order to confirm the above in vitro results, we then investigated mRNA and protein expression in tumours from 36th-, 38th-, 40th-, 43rd- and 46th-day ibuprofen-treated mice versus control using QPCR and western blot. Our results demonstrated that ICAM3, PDE3A, PRTN3, TRAF6, BCAR1, IL-1α, IL-1β, NFκB1, IκBκB, SOX2 and OCT4 mRNA expression decreases in the ibuprofen-treated groups versus control (Fig. 3e). In addition, we found that ICAM3, PDE3A, PRTN3, TRAF6, BCAR1, IL-1α, IL-1β, NFκB1, SOX2 and OCT4 protein expression similarly decreases in the ibuprofen-treated groups versus control (Fig. 3f) (CCL16 had no expression in mouse). The above-mentioned findings suggest that ibuprofen decreases the expression of inflammation-related stemness genes in vitro and in vivo.

### Ibuprofen mediates histone 3 methylation and acetylation to ICAM3 expression in vitro and in vivo

**Fig. 4:**
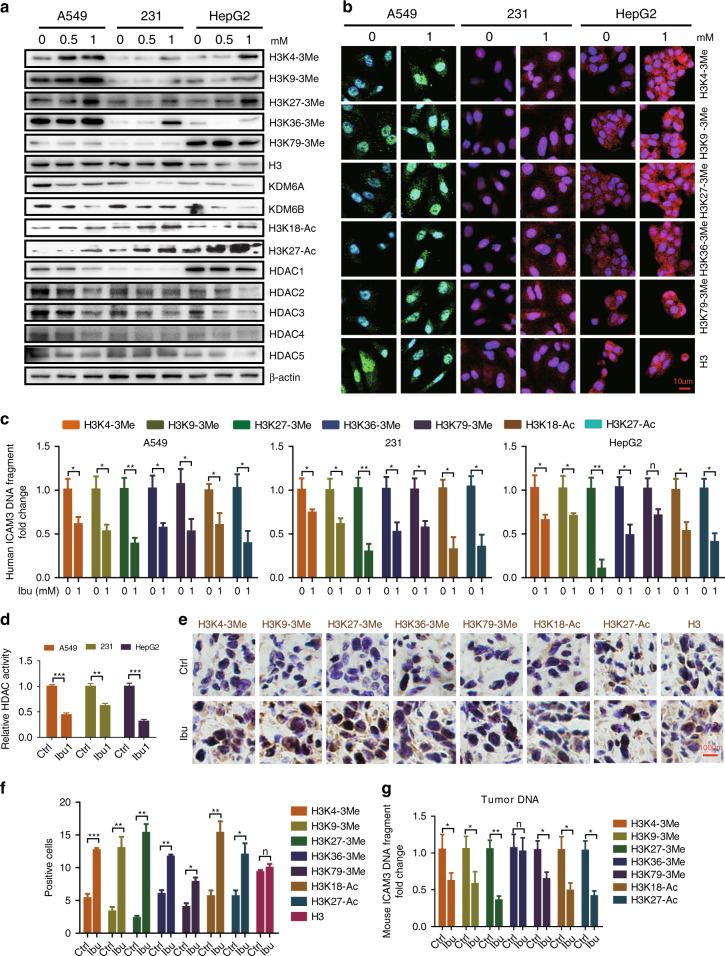
Ibuprofen mediates histone 3 methylation and acetylation to affect target genes’ expression in vitro and in vivo. **a** Western blot examining the expression of histone 3 modification markers, KDM6A, KDM6B and HDACs in A549, MDA-MB-231 and HepG2 cells with or without ibuprofen treatment. **b** Immunofluorescence staining of H3 modification markers in A549, MDA-MB-231 and HepG2 cells with or without ibuprofen treatment, scale bar: 10 µm. **c** CHIP-qPCR detecting the expression of ICAM3 DNA fragment in A549, MDA-MB-231 and HepG2 cells with or without ibuprofen treatment. **d** HDAC activity in A549, MDA-MB-231 and HepG2 cells with or without ibuprofen treatment. **e** Immunohistochemistry staining examining the expression of H3 modification markers in 4T1-luci tumours separated from BALB/c mice. Representative images with 40× magnification were shown. **f** The bar graph shows the statistic results of H3 modification marker-positive cells. **g** CHIP-qPCR detecting the expression of ICAM3 DNA fragment in 4T1-luci tumours separated from BALB/c mice with or without ibuprofen treatment. All data shown are representative of three experiment repeats.

In order to determine the mechanism underlying the action of ibuprofen, we explored the regulatory effect of ibuprofen on histone 3 modification markers in A549 lung cancer cells, MDA-MB-231 breast cancer cells and HepG2 liver cancer cells using western blot. Our results indicated that the expression of H3 trimethylation markers (i.e., H3K4–3Me, H3K9–3Me, H3K27–3Me, H3K36–3Me and H3K79–3Me) increases in the ibuprofen-treated groups versus control (Fig. 4a). We also found that the expression of histone demethylases (i.e., KDM6A and KDM6B) decreases in the ibuprofen-treated groups versus control (Fig. 4a). In addition, we found that the expression of H3 acetylation markers (i.e., H3K18-Ac and H3K27-Ac) increases in the ibuprofen-treated groups versus control (Fig. 4a). Since HDACs play a key role in regulating H3 acetylation, we determined the expression levels of HDACs using western blot. We found that the expression of HDAC 1–5 decreases in the ibuprofen-treated groups versus control (Fig. 4a). Accordingly, HDAC activity decreases in the ibuprofen-treated groups versus control (Fig. 4d).

To further ensure the regulatory effect of ibuprofen on histone 3 modifications in cancer stem cells and normal cancer cells, we detected that in ALDH + and ALDH− cells treated with ibuprofen by western blot. The results showed that the H3 modification markers were increased, the histone demethylases (KDM6A and KDM6B) and HDAC 1–5 decreased in both ALDH + and ALDH− cells (Supplementary Fig. S3B).

In order to verify the above findings, we studied the protein expression of H3K4–3Me, H3K9–3Me, H3K27–3Me, H3K36–3Me, H3K79–3Me and H3 in A549 lung cancer cells, MDA-MB-231 breast cancer cells and HepG2 liver cancer cells using immunofluorescence. Our results showed that the protein expression of the H3 modification markers within the nucleus increases in the ibuprofen-treated groups versus control (Fig. 4b).

In order to identify the role of H3 modification in regulating selected inflammation-related stemness genes, we measured the amount of ICAM3 DNA fragments in H3 modification marker pull-down DNAs in A549 lung cancer cells, MDA-MB-231 breast cancer cells and HepG2 liver cancer cells using the CHIP-qPCR assay. We selected ICAM3 since our previous studies demonstrated that ICAM mediates cancer cell inflammation and stemness. Our results demonstrated that the amount of ICAM3 DNA fragments in the various H3 modification marker pull-down DNAs decreases in all three cancer cell lines (Fig. 4c). The above-mentioned findings suggest that ibuprofen reduces histone demethylase (i.e., KDM6A and KDM6B) and HDAC expression that mediates histone 3 methylation and acetylation, and thereby inhibits gene expression.

In order to confirm the above in vitro results, we next examined H3 methylation and acetylation marker expression in tumours from ibuprofen-treated mice versus control using immunocytochemistry. Our results demonstrated that the H3 methylation and acetylation marker immunostaining within the nucleus increases in the ibuprofen-treated group versus control (Fig. 4e, f). We also found that the amount of ICAM3 DNA fragments in the various H3 modification marker pull-down DNAs decreases in the ibuprofen-treated group versus control, indicating that ICAM3 expression is blocked (Fig. 4g). These findings suggest that ibuprofen mediates H3 methylation and acetylation, and thereby regulates ICAM3 expression in vivo.

### Ibuprofen mediates H3 methylation and acetylation to regulate ICAM3 expression via a COX2-dependent manner

**Fig. 5:**
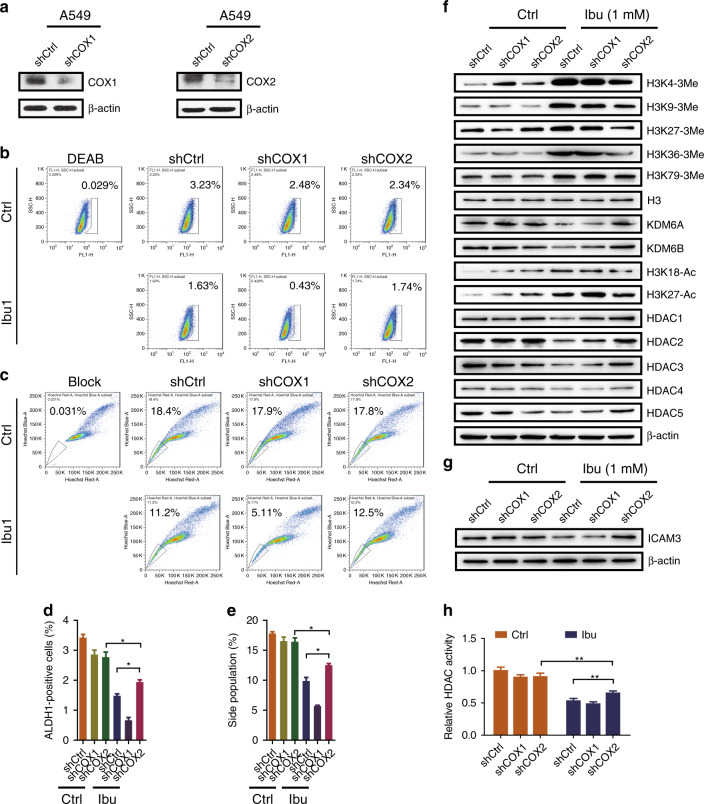
Ibuprofen mediates H3 methylation and acetylation to regulate ICAM3 expression in vitro. **a** Western blot examining the COX1- and COX2-knockdown efficiency in A549 cells. **b** ALDH staining assay was performed to check ALDH + sub-population percentage in A549-shCtrl, shCOX cells with or without ibuprofen treatment. **c** Side-population assay was performed to detect SP percentage in A549-shCtrl, shCOX cells with or without ibuprofen treatment. **d**, **e** Statistic results of ALDH + subpopulation and side population are shown. **f** Western blot examining the expression of histone 3 modification markers, KDM6A, KDM6B and HDACs in A549-shCtrl, shCOX cells with or without ibuprofen treatment. **g** HDAC activity in A549-shCtrl, shCOX cells with or without ibuprofen treatment. **h** Western blot examining the expression of ICAM3 in A549-shCtrl, shCOX cells with or without ibuprofen treatment. All data shown are representative of three experiment repeats.

In order to explore the role of COX in ibuprofen-mediated H3 methylation and acetylation, and targeted gene expression, we knocked down COX1 and COX2 expression in A549 cells, respectively (Fig. 5a) (knockdown efficiency 40–50%), then examined the ALDH + population, side population and chemoresistance. The results showed that the ALDH + population (Fig. 5b, d) and side population (Fig. 5c, e) were decreased in cells treated with ibuprofen. However, the ALDH + population and side population were partially rescued in shCOX2 cells compared with shCtrl cells treated with ibuprofen; the apoptosis was also rescued in shCOX2 cells compared with shCtrl cells treated with DDP and ibuprofen. We also found that the H3 trimethylation and triacetylation markers were increased, the histone demethylases (i.e., KDM6A and KDM6B) and HDAC (i.e., HDAC1,2,3) were decreased in cells treated with ibuprofen, but it could be rescued by knocking down COX2 treated with ibuprofen (Fig. 5f). Accordingly, HDAC activity was decreased in cells treated with ibuprofen versus control, and that was rescued in shCOX2 cells compared with shCtrl cells treated with ibuprofen (Fig. 5g). Moreover, as the new target genes, ICAM3 expression was decreased in cells treated with ibuprofen versus control, then that was rescued in shCOX2 cells compared with shCtrl cells treated with ibuprofen (Fig. 5h). These findings suggest that ibuprofen mediates H3 methylation and acetylation, and thereby regulates ICAM3 expression via a COX2-dependent manner.

### Ibuprofen combined with HDAC/HDM (KDM6A/B) inhibitors diminishes cancer progression in vivo

**Fig. 6:**
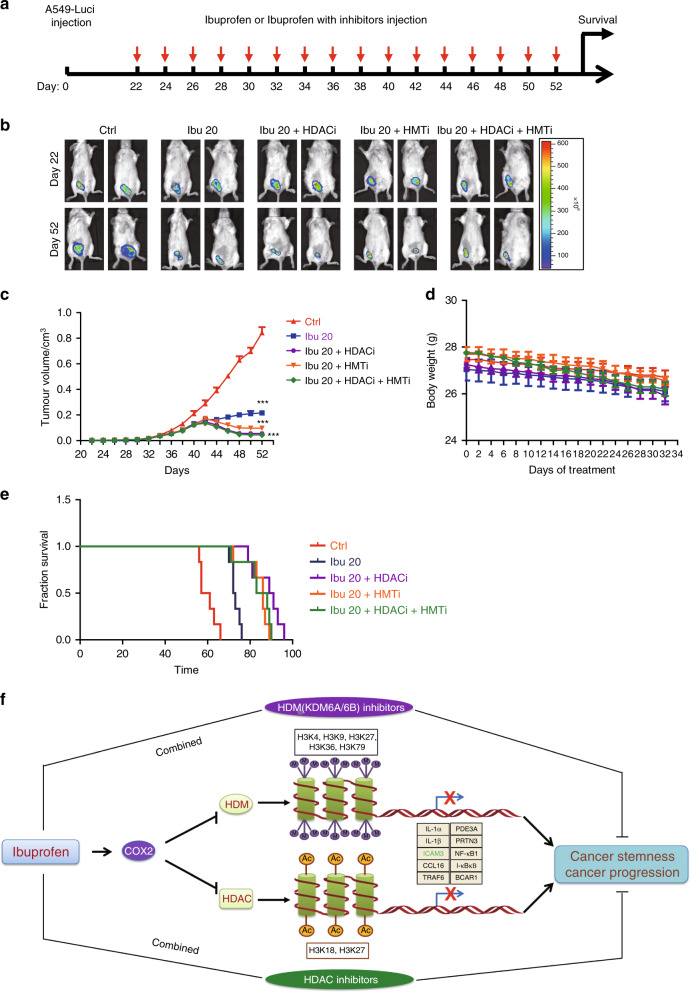
Ibuprofen combined HDAC/HDM (KDM6A/B) inhibitors that restrained cancer progression in vivo. **a** Schema of the inhibitor therapy model established by subcutaneous implantation of A549-luci cells into the NOD/SCID mice. **b** The representative luciferase images showing the A549-luci tumours at the primary sites of each group. **c** Tumour growth curve of A549-luci cells under combination therapy of ibuprofen and HDAC/HDM inhibitors. **d** The body weight of BALB/c mice inoculated with A549-luci cells under combination therapy of ibuprofen and HDAC/HDM inhibitors. **e** The survival curve of BALB/c mice inoculated with A549-luci cells under combination therapy of ibuprofen and HDAC/HDM inhibitors. **f** Proposed model of ibuprofen in mediating cancer cell stemness and cancer progression. All data shown are representative of three experiment repeats.

In order to investigate the use of ibuprofen combined with HDAC/HDM (KDM6A/B) inhibitors as a therapeutic strategy, we implanted A549-luceriferase cells into the fourth fat pad of male NOD/SCID mice. Twenty-three days after implantation, we injected IP the mice with 20 mg/kg ibuprofen, 20 mg/kg ibuprofen + 0.5 mg/kg HDAC inhibitor TSA, 20 mg/kg ibuprofen + 100 mg/kg KDM6A/B inhibitor (GSK J1), 20 mg/kg ibuprofen + 0.5 mg/kg HDAC inhibitor TSA + 100 mg/kg KDM6A/B inhibitor (GSK J1) or DMSO (control group) every 2 days (Fig. 6a). Our results showed that tumour size and volume decreased in the ibuprofen-treated group and the ibuprofen + inhibitor-treated groups versus DMSO control (Fig. 6b, c). However, we found that the body weight did not change significantly in the ibuprofen-treated group and the ibuprofen + inhibitor-treated groups versus DMSO control (Fig. 6d).

In addition, we found that the survival time increases in the ibuprofen-treated group and the ibuprofen + inhibitor-treated groups versus DMSO control (Fig. 6e). These results suggest that ibuprofen combined with HDAC/HDM (KDM6A/B) inhibitors diminishes cancer progression in vivo and may serve as a therapeutic strategy.

### Proposed model of ibuprofen in mediating cancer cell stemness and cancer progression

Based on our findings, we propose the following model (Fig. 6f). Ibuprofen inhibits histone demethylase (HDM) and HDAC expression that then mediates histone 3 methylation (H3K4–3Me, H3K9–3Me, H3K27–3Me, H3K36–3Me and H3K79–3Me) and acetylation (H3K18-Ac and H3K27-Ac), respectively. These H3 modifications then inhibit the expression of various inflammation-related stemness genes previously identified by high-throughput siRNA screening (IL-1α, IL-1β, ICAM3, CCL16, TRAF6, PDE3A, PRTN3, NFκB1, IκBκB and BCAR1). Using the ICAM3 gene as a representative of the inflammation-related stemness genes, ICAM3 expression is inhibited by the ibuprofen-mediated H3 modifications. Importantly, the above function of ibuprofen was mainly depending on COX2 expression. Thus, ibuprofen may diminish cancer cell stemness properties and cancer progression in vitro and in vivo by inhibiting the expression of various inflammation-related stemness genes with a COX2-dependent manner.

## Discussion

The anticancer potential of ibuprofen (a non-steroidal and anti-inflammatory drug) has created a broad interest to explore the clinical benefits of ibuprofen in cancer therapy.^29–31^ Previous findings by many investigators have established that ibuprofen induces apoptosis in cancer cells, and inhibits proliferation and metastasis of cancer cells.^11,12,32,33^ In addition, ibuprofen inhibits cancer stemness in gastric cancer although the mechanism of action remains unclear.^11^ In this study, we investigated the role of ibuprofen on cancer stemness in breast cancer, lung cancer and liver cancer. We found that ibuprofen diminishes cancer cell stemness properties that include reducing the ALDH + subpopulation, side population and sphere formation in all three cancer types in vitro. Also, ibuprofen inhibits tumour growth, metastasis, chemoresistance and prolongs survival in vivo. Our in vitro and in vivo studies reveal that the inhibitory role of ibuprofen occurs on multiple fronts in all three cancer types.

The well-characterised mechanism of action for ibuprofen involves the modification of Cox enzymes.^34,35^ In regard to ibuprofen in cancer therapy, an early report focused on the inhibition of the Cox-dependent pathway that leads to reduced inflammation and hence the anticancer properties of ibuprofen.^36^ Besides the inhibition of inflammation, it is not clear that other pathways or molecular mechanisms play a role in the anticancer properties of ibuprofen. In our study, we performed a more detailed analysis of the action of ibuprofen on histone modification. Our findings indicate that ibuprofen reduces HDACs and histone demethylase (KDM6A/B) expression that mediates histone acetylation and histone 3 methylation, and thereby suppresses inflammation-related stemness gene expression. Unexpectedly, the above process mainly depends on COX2 expression. However, we did not investigate the regulation of COX2 on HDAC or HDM6A/B in our present study. Collectively, these findings suggest a novel molecular mechanism that explains the anticancer properties of ibuprofen.

Cancer stem cells (CSCs) are a small population of cancer cells that possess the ability to self-renew, differentiate and modulate cancer growth, recurrence, metastasis and chemoresistance.^3,6,37,38^ The maintenance of cancer cell stemness largely depends on the surrounding inflammatory microenvironment.^39^ Our previous work established a medium- throughput siRNA screening platform to identify inflammation genes that regulate cancer cell stemness, and identified several novel candidates (e.g., ICAM3). ICAM3 mediates cancer cell stemness as well as cancer-related inflammation via Src/PI3K/AKT signalling.^6^ In our study, we clearly demonstrated that ibuprofen inhibits the expression of inflammation-related stemness genes (especially ICAM3). Moreover, ibuprofen mediates histone modification that causes an inhibition of inflammation-related stemness gene transcription that further suppresses cancer stemness. Our findings identify novel targets of ibuprofen that may explain the anticancer properties of ibuprofen, and may lead to new therapeutic strategies. In conclusion, our results demonstrated that ibuprofen diminishes cancer cell stemness properties and cancer progression in vitro and in vivo. Moreover, ibuprofen inhibits inflammation-related stemness gene expression, especially ICAM3 that we screened by high-throughput siRNA platform.

Our investigation of the underlying molecular mechanism demonstrated that ibuprofen reduces HDACs and histone demethylase (KDM6A/B) expression. This reduction mediates histone acetylation and histone 3 methylation, and thereby inhibits inflammation-related stemness gene expression. In regard to therapeutic strategies, ibuprofen combined with HDAC/HDM inhibitors diminished cancer progression in vivo. Therefore, our findings reveal a novel molecular mechanism that sheds further light on the anticancer properties of ibuprofen, and suggest therapeutic strategies for the prevention of cancer progression.

## Supplementary information

Supplemental information

## Data Availability

The data are available for all study authors. The data sets used and analysed during the current study are available from the corresponding author on reasonable request.

## References

[1] Jones DL, Wagers AJ (2008). No place like home: anatomy and function of the stem cell niche. Nat. Rev. Mol. Cell Biol..

[2] Prewitz MC, Seib FP, von Bonin M, Friedrichs J, Stissel A, Niehage C (2013). Tightly anchored tissue-mimetic matrices as instructive stem cell microenvironments. Nat. Methods.

[3] Visvader JE, Lindeman GJ (2012). Cancer stem cells: current status and evolving complexities. Cell. Stem Cell..

[4] van der Zee M, Sacchetti A, Cansoy M, Joosten R, Teeuwssen M, Heijmans-Antonissen C (2015). IL6/JAK1/STAT3 signaling blockade in endometrial cancer affects the ALDHhi/CD126+ stem-like component and reduces tumor burden. Cancer Res..

[5] Yang J, Liao D, Chen C, Liu Y, Chuang TH, Xiang R (2013). Tumor-associated macrophages regulate murine breast cancer stem cells through a novel paracrine EGFR/Stat3/Sox-2 signaling pathway. Stem Cells.

[6] Shen W, Xie J, Zhao S, Du R, Luo X, He H (2018). ICAM3 mediates inflammatory signaling to promote cancer cell stemness. Cancer Lett..

[7] Schack A., Fransgaard T., Klein M. F. & Gogenur I. Perioperative use of nonsteroidal anti-inflammatory drugs decreases the risk of recurrence of cancer after colorectal resection: a cohort study based on prospective data. *Ann. Surg Oncol*. **26**, 3826–3837 (2019). 10.1245/s10434-019-07600-8 31313040

[8] Kehm RD, Hopper JL, John EM, Phillips KA, MacInnis RJ, Dite GS (2019). Regular use of aspirin and other non-steroidal anti-inflammatory drugs and breast cancer risk for women at familial or genetic risk: a cohort study. Breast Cancer Res..

[9] Pennock ND, Martinson HA, Guo Q, Betts CB, Jindal S, Tsujikawa T (2018). Ibuprofen supports macrophage differentiation, T cell recruitment, and tumor suppression in a model of postpartum breast cancer. J. Immunother. Cancer.

[10] Liu X, Wang X, Zhao W, Wei L, Zhang P, Han F (2018). A prospective, randomized, double-blind, placebo-controlled trial of acute postoperative pain treatment using opioid analgesics with intravenous ibuprofen after radical cervical cancer surgery. Sci. Rep..

[11] Akrami H, Moradi B, Borzabadi Farahani D, Mehdizadeh K (2018). Ibuprofen reduces cell proliferation through inhibiting Wnt/beta catenin signaling pathway in gastric cancer stem cells. Cell Biol. Int..

[12] Stabile LP, Farooqui M, Kanterewicz B, Abberbock S, Kurland BF, Diergaarde B (2018). Preclinical evidence for combined use of aromatase inhibitors and NSAIDs as preventive agents of tobacco-induced lung cancer. J. Thorac. Oncol..

[13] Ait Ouakrim D, Dashti SG, Chau R, Buchanan DD, Clendenning M, Rosty C (2015). Aspirin, ibuprofen, and the risk of colorectal cancer in lynch syndrome. J. Natl Cancer Inst.

[14] Becker C, Wilson JC, Jick SS, Meier CR (2015). Non-steroidal anti-inflammatory drugs and the risk of head and neck cancer: a case-control analysis. Int. J. Cancer.

[15] Khan MN, Lee YS (2011). Cyclooxygenase inhibitors: scope of their use and development in cancer chemotherapy. Med. Res. Rev..

[16] Qadri SS, Wang JH, Redmond KC, AF OD, Aherne T, Redmond HP (2002). The role of COX-2 inhibitors in lung cancer. Ann. Thorac. Surg..

[17] Bannister AJ, Kouzarides T (2011). Regulation of chromatin by histone modifications. Cell Res..

[18] Zou C, Mallampalli RK (2014). Regulation of histone modifying enzymes by the ubiquitin-proteasome system. Biochim Biophys. Acta.

[19] Kondo Y (2009). Epigenetic cross-talk between DNA methylation and histone modifications in human cancers. Yonsei Med. J..

[20] Greer EL, Shi Y (2012). Histone methylation: a dynamic mark in health, disease and inheritance. Nat. Rev. Genet..

[21] Guo Y, Liu Y, Zhang C, Su ZY, Li W, Huang MT (2016). The epigenetic effects of aspirin: the modification of histone H3 lysine 27 acetylation in the prevention of colon carcinogenesis in azoxymethane- and dextran sulfate sodium-treated CF-1 mice. Carcinogenesis.

[22] Son DS, Wilson AJ, Parl AK, Khabele D (2010). The effects of the histone deacetylase inhibitor romidepsin (FK228) are enhanced by aspirin (ASA) in COX-1 positive ovarian cancer cells through augmentation of p21. Cancer Biol. Ther..

[23] Chen Z, Li W, Qiu F, Huang Q, Jiang Z, Ye J (2018). Aspirin cooperates with p300 to activate the acetylation of H3K9 and promote FasL-mediated apoptosis of cancer stem-like cells in colorectal cancer. Theranostics.

[24] Zhao S, Shen W, Yu J, Wang L (2018). TBX21 predicts prognosis of patients and drives cancer stem cell maintenance via the TBX21-IL-4 pathway in lung adenocarcinoma. Stem Cell Res. Ther..

[25] Shen W, Zhang X, Du R, Fan Y, Luo D, Bao Y (2018). ICAM3 mediates tumor metastasis via a LFA-1-ICAM3-ERM dependent manner. Biochim Biophys. Acta.

[26] Shen W, Chang A, Wang J, Zhou W, Gao R, Li J (2015). TIFA, an inflammatory signaling adaptor, is tumor suppressive for liver cancer. Oncogenesis.

[27] Shen W, Du R, Li J, Luo X, Zhao S, Chang A (2016). TIFA suppresses hepatocellular carcinoma progression via MALT1-dependent and -independent signaling pathways. Signal Transduct. Target Ther..

[28] Luo DH, Zhang XY, Du RL, Gao WJ, Luo N, Zhao ST (2018). Low dosage of arsenic trioxide (As2O3) inhibits angiogenesis in epithelial ovarian cancer without cell apoptosis. J. Biol. Inorg. Chem..

[29] Moon HJ, Kim HB, Lee SH, Jeun SE, Kang CD, Kim SH (2018). Sensitization of multidrug-resistant cancer cells to Hsp90 inhibitors by NSAIDs-induced apoptotic and autophagic cell death. Oncotarget.

[30] Dandah O, Najafzadeh M, Isreb M, Linforth R, Tait C, Baumgartner A (2018). Aspirin and ibuprofen, in bulk and nanoforms: Effects on DNA damage in peripheral lymphocytes from breast cancer patients and healthy individuals. Mutat. Res. Genet. Toxicol. Environ. Mutagen..

[31] Shi J, Leng W, Zhao L, Xu C, Wang J, Chen X (2017). Nonsteroidal anti-inflammatory drugs using and risk of head and neck cancer: a dose-response meta analysis of prospective cohort studies. Oncotarget.

[32] Tran BN, Nguyen HT, Kim JO, Yong CS, Nguyen CN (2017). Combination of a chemopreventive agent and paclitaxel in CD44-targeted hybrid nanoparticles for breast cancer treatment. Arch. Pharm. Res..

[33] Lima RA, Candido EB, de Melo FP, Piedade JB, Vidigal PV, Silva LM (2015). Gene expression profile of ABC transporters and cytotoxic effect of ibuprofen and acetaminophen in an epithelial ovarian cancer cell line in vitro. Rev. Bras. Ginecol. Obstet..

[34] Simmons DL, Botting RM, Hla T (2004). Cyclooxygenase isozymes: the biology of prostaglandin synthesis and inhibition. Pharm. Rev..

[35] Mandal P, Kundu BK, Vyas K, Sabu V, Helen A, Dhankhar SS (2018). Ruthenium(ii) arene NSAID complexes: inhibition of cyclooxygenase and antiproliferative activity against cancer cell lines. Dalton Trans..

[36] Afzal M, Kazmi I, Khan R, Rana P, Kumar V, Al-Abbasi FA (2017). Thiamine potentiates chemoprotective effects of ibuprofen in DEN induced hepatic cancer via alteration of oxidative stress and inflammatory mechanism. Arch. Biochem. Biophys..

[37] Magee JA, Piskounova E, Morrison SJ (2012). Cancer stem cells: impact, heterogeneity, and uncertainty. Cancer Cell..

[38] Nassar D, Blanpain C (2016). Cancer stem cells: basic concepts and therapeutic implications. Annu. Rev. Pathol..

[39] Hanahan D, Weinberg RA (2011). Hallmarks of cancer: the next generation. Cell.

