# Supplementary material for: Ibuprofen mediates histone modification to diminish cancer cell stemness properties via a COX2-dependent manner
Source: Br J Cancer. 2020 Jun 12;123(5):730–41. doi: 10.1038/s41416-020-0906-7 (PMC7463005; doi:10.1038/s41416-020-0906-7)
Supplement: Supplementary file 1 — Supplemental information [file 41416_2020_906_MOESM1_ESM.pdf]

Supplemental Table 1. Primer sequences

Name	Sequence
ICAM3-H-RT-F	CCCCAGCACTTGAAATGGAAAGA
ICAM3-H-RT-R	AGGGTCAGTAACACCGCCACGAA
CCL16-RT-F	CTTATCATTACTTCGGCTTCTCGC
CCL16-RT-R	GGCCTTTCTGTATCCCACCACTA
PDE3A-RT-F	GATGATAAATACGGATGTCTGTCTGG
PDE3A -RT-R	GACAAGGAAACGGAAATGCTTAA
PRTN3-RT-F	ACAACTACGACGCGGAGAACAAACTGA
PRTN3-RT-R	GAAGAAGGTGACCACGGTGACATTGAG
TRAF6-RT-F	GCACGCCACCTACAAGAG
TRAF6-RT-R	CAGGGCTATGAATCACAACA
BCAR1-RT-F	CAGTTTGAACGACTGGAACAGGAGGTG
BCAR1-RT-R	AGCTTGTGGGCGCTGAGGATGAC
IL-1α-RT-F	ATTGTATGTGACTGCCCAAGATG
IL-1α-RT-R	GTTTCCCAGAAGAAGAGGAGGTT
IL-1β-RT-F	GGCGGCATCCAGCTACGAATCTC
IL-1β-RT-R	AAGGTCTGTGGGCAGGGAACCAG
NF-κB1-RT-F	AAAAGGACCCTGAAGGTTGTGAC
NF-κB1-RT-R	AAGGTGGATGATTGCTAAGTGTAAG

IκBκB-RT-F	GGCAGTCTTTGCACATCATTCGT
IκBκB-RT-R	TCACCGTTCCATTCAAGTCTTCG
SOX2-RT-F	TGGAGCAACGGCAGCTACAGCATG
SOX2-RT-R	GGAGTGGGAGGAAGAGGTAACCACAGG
OCT4-RT-F	TGGGAAGGTATTCAGCCAAACGA
OCT4-RT-R	ACCGAGGAGTACAGTGCAGTGAAGTGA
ICAM3-M-RT-F	GCAGAACAGGAAGGCACCAAACAG
ICAM3-M-RT-R	CTCAAAGTCAGAAGAGGAGTCGGGAAG
ICAM3-CHIP-3-F	CTTGCACAGGAACAGTAGCG
ICAM3-CHIP-3-R	ACGAAGAACGGGATCCC
COX1-sh	AAAAGGAGTACAGCTACGAGCAGTTTTGGATCCAAAACTGCTCGTAGCTGTACTCC
COX2-sh	AAAAGCGCTCAGCCATACAGCAAATTTGGATCCAAATTTGCTGTATGGCTGAGCGC

Supplemental Table 2. Antibodies List

Antibody		Clone, Cat #	Vendor	City, State, Country
ICAM3	Rabbit monoclonal	EPR3994, ab109405	Abcam	Hong Kong, China
CCL16	Rabbit monoclonal	EPR4452(2), ab134917	Abcam	Hong Kong, China
PDE3A	Rabbit monoclonal	EPR11601, ab169534	Abcam	Hong Kong, China

PRTN3	Rabbit monoclonal	EPR6227, ab133613	Abcam	Hong Kong, China
IL-1α	Rabbit polyclonal	ab9614	Abcam	Hong Kong, China
OCT4	Rabbit polyclonal	ab19857	Abcam	Hong Kong, China
NANOG	Rabbit monoclonal	ab109250	Abcam	Hong Kong, China
ALDH1A1	Rabbit monoclonal	ab52492	Abcam	Hong Kong, China
SOX2	Rabbit polyclonal	H-65, sc-20088X	Santa Cruz Biotechnology	Santa Cruz, CA, USA
TRAF6	Rabbit polyclonal	H-274, sc-7221	Santa Cruz Biotechnology	Santa Cruz, CA, USA
BCAR1	Rabbit polyclonal	C-20, sc-860	Santa Cruz Biotechnology	Santa Cruz, CA, USA
β-actin	Mouse monoclonal	sc-47778	Santa Cruz Biotechnology	Santa Cruz, CA, USA
IL-1β	Mouse monoclonal	12242	Cell Signal Technology	Danvers, MA, USA
p-IKKβ	Mouse monoclonal	16A6, 2697L	Cell Signal Technology	Danvers, MA, USA
H3K4-3Me	Rabbit monoclonal	9783 (Kit)	Cell Signal Technology	Danvers, MA, USA
H3K9-3Me	Rabbit monoclonal	9783 (Kit)	Cell Signal Technology	Danvers, MA, USA
H3K27-3Me	Rabbit monoclonal	9783 (Kit)	Cell Signal Technology	Danvers, MA, USA
H3K36-3Me	Rabbit monoclonal	9783 (Kit)	Cell Signal Technology	Danvers, MA, USA
H3K79-3Me	Rabbit monoclonal	9783 (Kit)	Cell Signal Technology	Danvers, MA, USA
H3	Rabbit monoclonal	9783 (Kit)	Cell Signal Technology	Danvers, MA, USA
H3K18-Ac	Rabbit monoclonal	9927 (Kit)	Cell Signal Technology	Danvers, MA, USA
H3K27-Ac	Rabbit monoclonal	9927 (Kit)	Cell Signal Technology	Danvers, MA, USA
HDAC1	Mouse monoclonal	9928 (Kit)	Cell Signal Technology	Danvers, MA, USA
HDAC2	Mouse monoclonal	9928 (Kit)	Cell Signal Technology	Danvers, MA, USA
HDAC3	Mouse monoclonal	9928 (Kit)	Cell Signal Technology	Danvers, MA, USA

HDAC4	Rabbit monoclonal	9928 (Kit)	Cell Signal Technology	Danvers, MA, USA
HDAC5	Mouse monoclonal	9928 (Kit)	Cell Signal Technology	Danvers, MA, USA
KDM6A	Rabbit monoclonal	33510	Cell Signal Technology	Danvers, MA, USA
KDM6B	Rabbit monoclonal	3457	Cell Signal Technology	Danvers, MA, USA
COX1	Rabbit monoclonal	9896	Cell Signal Technology	Danvers, MA, USA
COX2	Rabbit monoclonal	12282	Cell Signal Technology	Danvers, MA, USA

Supplemental figure 1

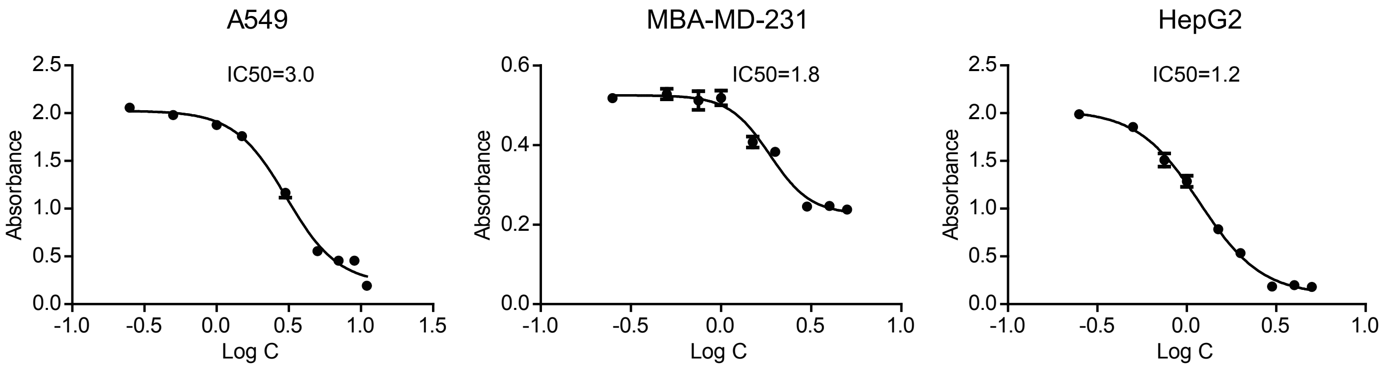

Fig. S1 The work concentration of ibuprofen was tested in various cancer cells.

The cytotoxicity assay was performed on lung cancer cell A549, breast cancer cell MDA-MB-231and liver cancer cell HepG2. The IC50 of ibuprofen on three cell lines were 3.0, 1.8, 1.2 respectively.

Supplemental figure 2

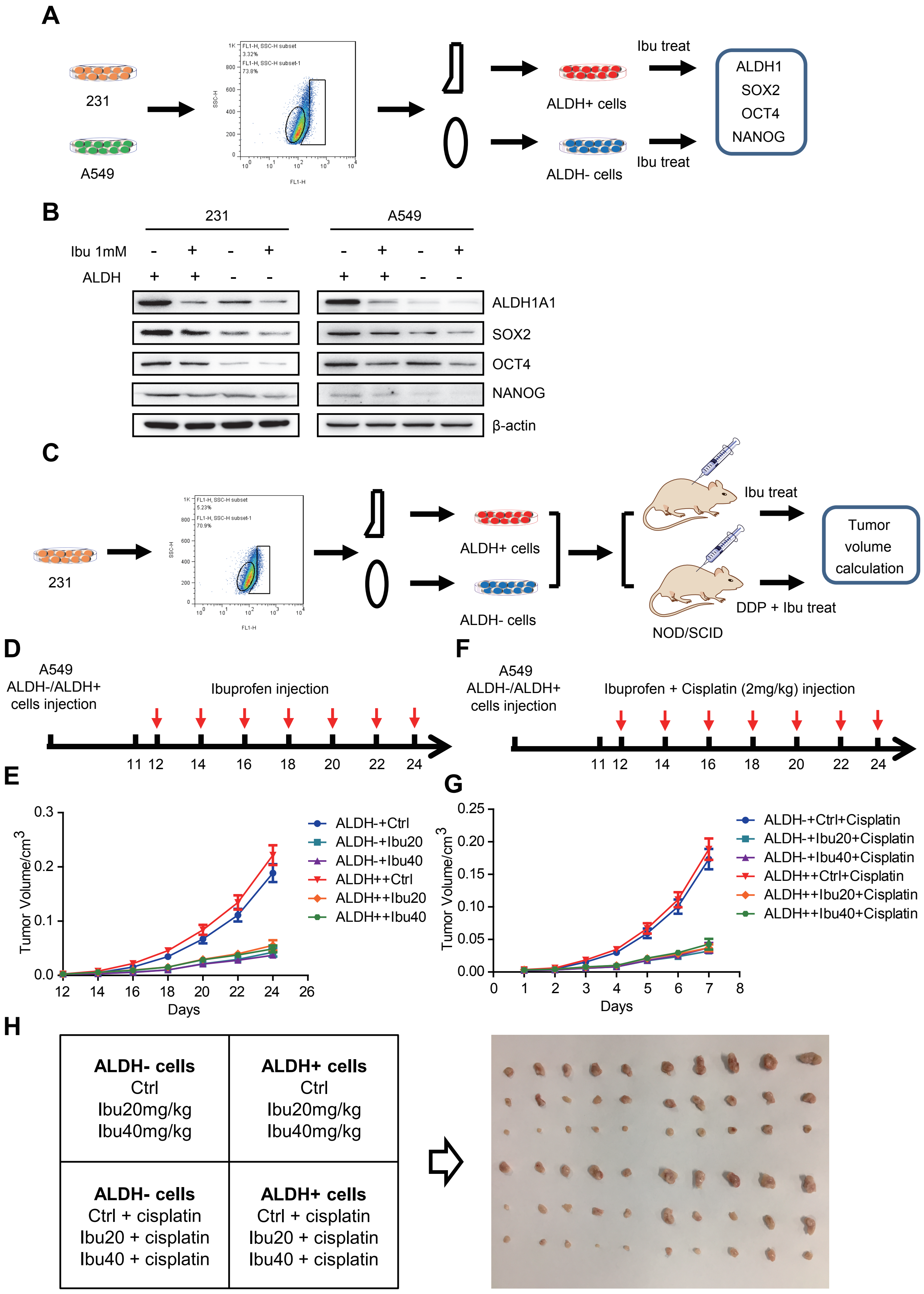

Fig. S2 The effects of ibuprofen on CSC or non-CSC cells was detected.

(A) Schematic model of CSC or non-CSC cell sorting. (B) Western blot to detect the stemness markers (ALDH1A1, SOX2, OCT4 and NANOG) in ALDH+ or ALDH- cells treated with ibuprofen sorted from 231 and A549 cell lines. (C) Schematic model of the *in vivo* experiment to check the role of ibuprofen on CSC or non-CSC cells. (D) Schema of the *in vivo* model established by subcutaneous implantation of ALDH+/ALDH- cells into the 4th pair of mammary fat pad of nude mice. (E) Tumor growth curve of ALDH+/ALDH- cells with or without ibuprofen treatment. (F) Schema of the chemo-resistance model established by subcutaneous implantation of ALDH+/ALDH- cells into the 4th pair of mammary fat pad of nude mice. (G) Tumor growth curve of ALDH+/ALDH- cells with or without ibuprofen treatment in the presence of cisplatin. (H) The representative tumors separated from each groups were shown.

Supplemental figure 3

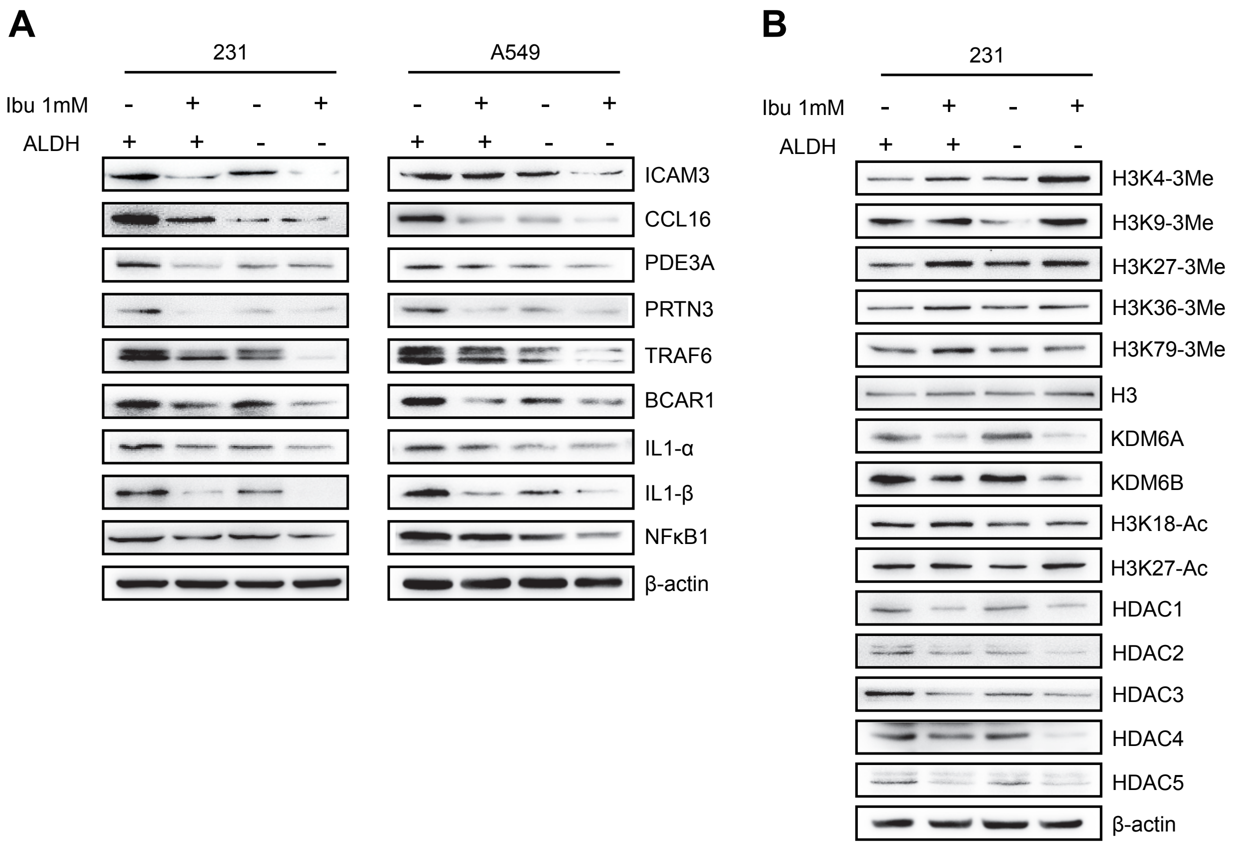

Fig. S3 Ibuprofen effects the inflammation related genes and H3 modification markers expression in ALDH+ and ALDH- cells.

(A) Western blot to detect the inflammation related genes expression in ALDH+ and ALDH- cells with or without ibuprofen treatment. (B) Western blot to detect the H3 modification markers expression in ALDH+ and ALDH- cells with or without ibuprofen treatment.
